# Special Issue—Towards Understanding the Mechanisms and Curing of Muscular Dystrophy Diseases

**DOI:** 10.3390/molecules200712944

**Published:** 2015-07-16

**Authors:** Leonidas A. Phylactou

**Affiliations:** The Cyprus Institute of Neurology & Genetics, PO Box 23462, 1683 Nicosia, Cyprus; E-Mail: laphylac@cing.ac.cy

Muscular dystrophies are a heterogeneous group of inherited diseases with different molecular basss, but sharing similar clinical features and dystrophic changes. Understanding disease mechanisms for muscular dystrophies and, moreover, finding efficient and permanent ways of curing them remains challenging. The purpose of this issue was to include manuscripts which aim at addressing the understanding of disease mechanisms in muscular dystrophies and ways to tackle them. Two reviews and three original articles compose the current Special Issue [1,2,3,4,5]. Two of the articles describe new methods for the identification of biomarkers in Duchenne Muscular Dystrophy [1,2]; a non-invasive method in the sera of patients [1] and a muscle-specific approach in the known mdx animal model [2]. Moreover, a paper in this Special Issue describes how cell culture conditions affect gene expression in Facioscapulohumeral muscular dystrophy myoblasts [3]. Finally, three reviews describe, in a very comprehensive way, ways to tackle muscular dystrophy through model organisms [4], and the current understanding of the pathology and treatment of cardiomyopathy in Duchenne Muscular Dystrophy [5,6].
